# Genetic diversity and mate selection in a reintroduced population of gray wolves

**DOI:** 10.1038/s41598-021-04449-4

**Published:** 2022-01-11

**Authors:** David E. Ausband

**Affiliations:** grid.266456.5 0000 0001 2284 9900 U.S. Geological Survey, Idaho Cooperative Fish and Wildlife Research Unit, University of Idaho, 875 Perimeter Drive, MS 1141, Moscow, ID 83844 USA

**Keywords:** Ecology, Genetics

## Abstract

The genetic composition of an individual can markedly affect its survival, reproduction, and ultimately fitness. As some wildlife populations become smaller, conserving genetic diversity will be a conservation challenge. Many imperiled species are already supported through population augmentation efforts and we often do not know if or how genetic diversity is maintained in translocated species. As a case study for understanding the maintenance of genetic diversity in augmented populations, I wanted to know if genetic diversity (i.e., observed heterozygosity) remained high in a population of gray wolves in the Rocky Mountains of the U.S. > 20 years after reintroduction. Additionally, I wanted to know if a potential mechanism for such diversity was individuals with below average genetic diversity choosing mates with above average diversity. I also asked whether there was a preference for mating with unrelated individuals. Finally, I hypothesized that mated pairs with above average heterozygosity would have increased survival of young. Ultimately, I found that females with below average heterozygosity did not choose mates with above average heterozygosity and wolves chose mates randomly with respect to genetic relatedness. Pup survival was not higher for mated pairs with above average heterozygosity in my models. The dominant variables predicting pup survival were harvest rate during their first year of life and years pairs were mated. Ultimately, genetic diversity was relatively unchanged > 20 years after reintroduction. The mechanism for maintaining such diversity does not appear related to individuals preferentially choosing more genetically diverse mates. Inbreeding avoidance, however, appears to be at least one mechanism maintaining genetic diversity in this population.

## Introduction

The genetic composition of an individual can markedly affect its survival, reproduction, and ultimately fitness^1^. Because they are often coupled with conservation efforts, many studies have focused on species that are genetically impoverished (e.g., low individual heterozygosity^2–4^). From a wealth of such studies, we know that being genetically impoverished can lead to changes in mating behaviors (e.g., time spent mating^5^), reduced reproductive vital rates^6^, an inability to combat disease^7^, reduced survival, and increased probability of population extinction^8^. In contrast, having a diverse genetic composition can lead to increased fitness over a lifetime^9^. Indeed, correlations between individual heterozygosity and fitness (i.e., heterozygosity-fitness correlations) have been observed in several species^1^ although the overall strength of the effect has been questioned^10,11^. Selecting for genetically diverse mates is not the only path to enhanced diversity, however, individuals may also preferentially choose to mate with unrelated individuals (i.e., inbreeding avoidance) making loss of genetic diversity less likely over time.

For mammals, both sexes breed and the young they produce are diploid, receiving half of their DNA from each parent. Selection may favor individuals who, if genetically impoverished, choose to breed with more diverse partners so that their resulting young are more genetically diverse than the parents^12^. Indeed, studies have shown that individuals will choose mates with higher average heterozygosity than what is expected if there were random mating in the population^13,14^.

As many wildlife populations become smaller, fragmented, and isolated, conserving genetic diversity will be a conservation challenge. For example, many imperiled species are supported through population augmentation efforts such as captive breeding and translocations. Many augmentation efforts actively manage for genetic diversity^15^ although in some cases there are few remaining individuals and managers are limited by an inherent lack of remnant diversity^16,17^. Even when the best efforts are made and sufficiently diverse source populations remain, the ability of managers to maintain diversity in wild populations hinges on largely uncontrollable mate-selection choices made by resident or translocated individuals. We often do not know how genetic diversity is maintained in the wild after release for many translocated species.

Gray wolves (*Canis lupus*) were extirpated from the Rocky Mountains of the U.S. by the 1930s. A small (n = 35), genetically diverse (Observed heterozygosity, H_o_ = 0.70^18^) population of wolves was reintroduced to Idaho, USA, in 1995–1996 from nearby source populations in Alberta and British Columbia, Canada^19^. Wolves are generally considered quite fecund, but their social structure typically limits breeding to two individuals per group, thus the effective population size or number of breeding individuals is a small fraction of total population abundance. Although typically monogamous, wolf mating systems are flexible with polygyny, polyandry, and “sneaker” males observed in wild populations^20^. Rubenstein^14^ found such extrapair matings in some bird populations were a means to secure more genetically diverse offspring although this has yet to be explored in wolves.

I wanted to know if genetic diversity remained high in a population > 20 years after reintroduction. Therefore, I evaluated (1) genetic diversity in a reintroduced population of gray wolves, (2) if genetic diversity was maintained in this population through individuals with below average genetic diversity choosing mates with above average genetic diversity, (3) if wolves showed inbreeding avoidance by mating with unrelated individuals, and (4) if mated pairs with above average heterozygosity had increased survival of young.

## Materials and methods

### Study areas

**Figure 1:**
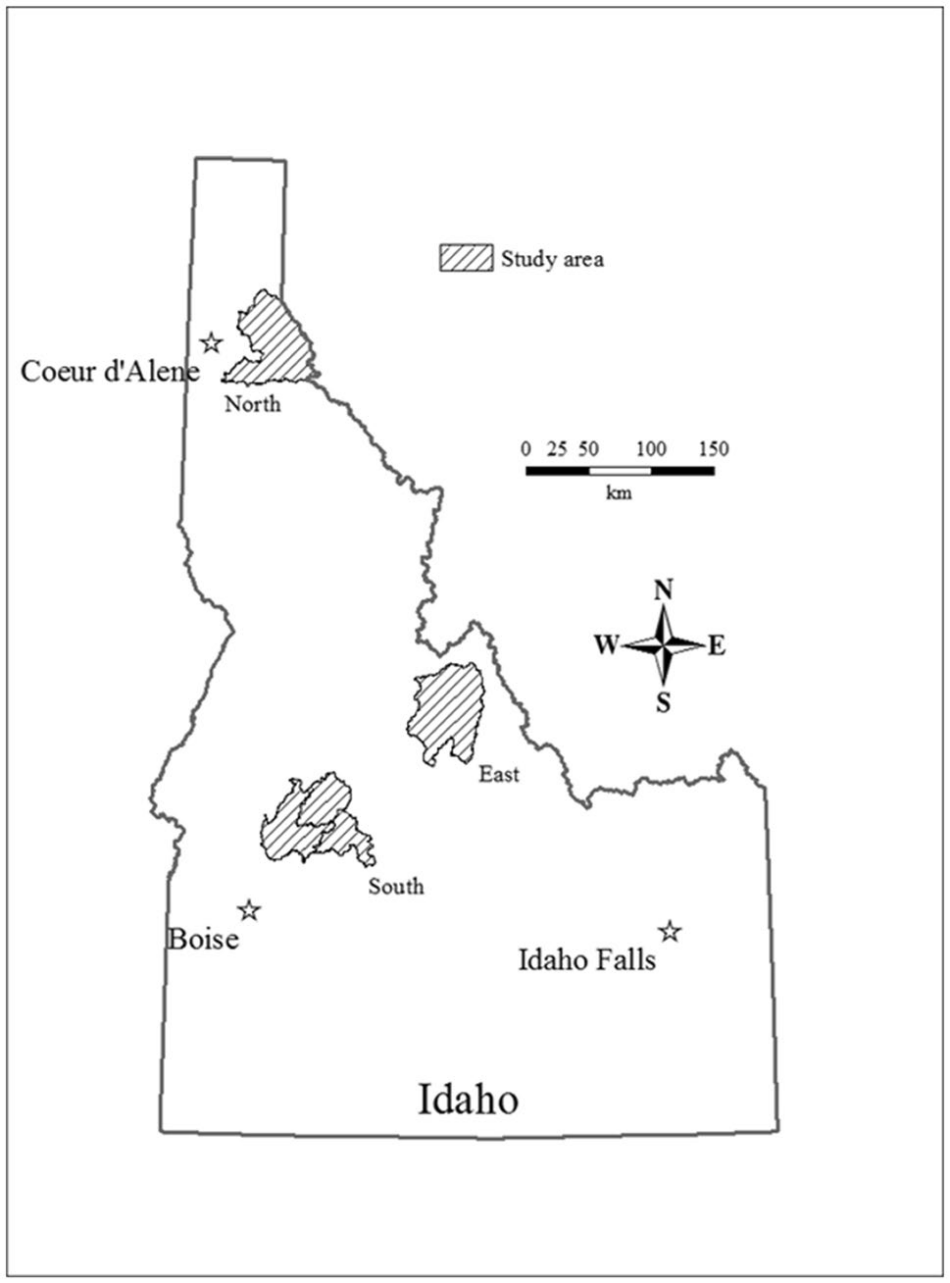
Study areas (i.e., subpopulations) in Idaho, USA, where wolves were genetically sampled, 2008–2017.

I sampled wolves in three study areas (north, east, and south) that included 5 Game Management Units (GMU’s) within Idaho, USA (GMUs 4, 28, 33–35; Fig. 1). Each study area was embedded in the larger population of wolves that spanned most of Idaho and movement of wolves from one study area to another was rare due to distance (Fig. 1). All study areas were mountainous regions of primarily United States Forest Service (USFS) lands. During the years we sampled, annual temperatures ranged from − 13 °C to 36 °C^21^. Annual precipitation ranged from 30 to 130 cm^21^. Elevation ranged from 646 to 3219 m. The northern study area (GMU 4; 3189 km^2^) had a maritime climate dominated by western red cedar (*Thuja plicata*), Douglas fir (*Pseudotsuga menziesii*), Engelmann spruce (*Picea engelmannii*), and lodgepole pine (*Pinus contorta*). The eastern (GMU 28; 3388 km^2^) and southern (GMUs 33–35; 3861 km^2^) study areas had a continental climate and were dominated by ponderosa pine (*P. ponderosa*), lodgepole pine, spruce mixed forests, and sagebrush (*Artemisia tridentata*) steppe. Public harvest of wolves began in Idaho in 2009, temporarily ceased in 2010, and began again in 2011 and annually thereafter. Wolves were harvested using rifles (65.7%), traps (32.7%), and archery (< 2%)^22^. Most harvest occurred during September–March with a peak during the big-game rifle hunting season in October–November^22^.

### Field methods

From June to August 2008–2017, we collected wolf scats at occupied or recently occupied wolf pup-rearing sites. When available, we used GPS or radio-telemetry locations of wolves to locate pup-rearing sites. During 2008–2010 we used no radiocollar information to find wolves, from 2011 to 2014 approximately 65% of our study groups contained collared individuals although their locations did not always help us find pups. From 2015 to 2018, we again used no collar information. At sites of both collared and uncollared wolves, we surveyed historical pup-rearing sites and sites predicted by a habitat model to have a high probability (≥ 70% suitability) of being a pup-rearing site^23^. At each predicted site, technicians attempted to find an activity center (area where pups congregate) through howl surveys^24^ or by searching the area around wolf GPS clusters and radio-telemetry locations. Once the activity center was located, 4–8 technicians collected pup and adult scat for 3–5 h, radiating out from the activity center up to 500 m to ensure a sufficient sample of adult scats^23,25^. Incidental scats found outside of pup-rearing sites were also collected. We collected 125–200 samples per group per year, which generally detected every individual in the group^25^. We attempted to resample each group (n = 16) in every study area every year.

### Laboratory methods

DNA analyses were conducted at the University of Idaho’s Laboratory for Ecological, Evolutionary and Conservation Genetics (Moscow, ID, USA). We extracted DNA from scats using Qiagen kits (DNA stool mini kit and DNeasy; Qiagen, Valencia, CA, USA) with a negative control to test for contamination. We first screened all samples in a species-identification test using co-amplification and fragment analysis of 3 short segments of the mitochondrial DNA control region to remove non-target species and low-quality samples^26^. We then attempted to genotype all samples identified during the mtDNA test as wolf or dog (*C. familiaris*) using 18 nuclear DNA microsatellite loci; (AHT103, AHT109, AHT121, AHT200, C05.377, C09.173, C37.172, Cxx.119, Cxx.250, FH2001, FH2004, FH2010, FH2054, FH2088, FH2137, FH2611, FH2670, FH3725)^27–31^. We initially amplified all samples twice for genotyping and required successful amplification of alleles at ≥ 5 loci for the sample to continue for an additional 1–3 PCRs, whereas we discarded samples that amplified at < 5 loci. For each locus, we required ≥ 2 independent PCR amplifications for consensus of a heterozygote and ≥ 3 independent PCR amplifications for consensus of a homozygote. We compared all consensus genotypes (i.e., 18 loci) and all unique genotypes of previously identified individuals using Genalex 6.503^32^ to match samples and distinguish unique genotypes. To avoid overestimation and account for undetected genotyping errors, we grouped samples mismatching by allelic dropout at only 1 locus (e.g., 102, 0 vs. 102, 106) as a single individual^33^. We used Reliotype^34^ to test the accuracy of unique genotypes represented by only 1 noninvasive sample (i.e., single detections) by ensuring the genotype attained a 95% accuracy threshold. Further details regarding laboratory methods can be found in^25,35–37^.

In 2008 and 2009, we analyzed all collected samples. After 2010, we analyzed 40 adult and 25 pup samples from each group based on results of resampling and rarefaction^35^. If a group had more than two individuals detected only once we analyzed additional samples when available to obtain 10 more consensus genotypes. We compared genotypes from tissue samples of harvested wolves in fall/winter to genotypes of wolves detected in the previous summer by fecal DNA sampling to estimate an annual harvest rate (i.e., number detected in harvest/number detected in summer).

## Analyses

For each year I included all sampled adult males and females as potential parents and all sampled pups as potential offspring of every pair of individuals and then determined breeders and their offspring by constructing pedigrees using maximum-likelihood in program COLONY version 2.0.5.5^38^. I first calculated allele frequencies for each year in program COANCESTRY version 1.0.1.5^39^ and then imported the frequencies into program COLONY for use in pedigree analyses. I allowed for polygamy in both males and females and assumed an allelic dropout rate of 0.01 and genotyping error rate of 0.01. In cases where parentage was undetermined from COLONY, I further examined offspring genotypes against the likely parents of the remaining offspring in the group and allowed for a two allele mismatch owing to allelic dropout between parent and offspring to verify parentage across the 18 loci using exclusion methods^1^. Once breeders were identified from pedigree analyses, I used Program Coancestry^39^ to estimate Trio ML genetic relatedness between the breeders. Lastly, I used Program STORM^40^ to assess whether relatedness between observed breeding pairs was different if there were random mating in the population. To do this, I populated STORM with 151 potentially reproductive (≥ 2 years old) male genotypes and 167 potential reproductive (≥ 2 years old) female genotypes. I then simulated 151 mated pairs using sampling with replacement and estimated relatedness between simulated mated pairs over 10,000 iterations.

I sampled the same groups of wolves across consecutive years and from the resulting pedigrees determined the breeders, pups, paternity from sneaker males (i.e., males who sired pups but were unsampled and unaffiliated with any sampled pack), evidence of polygamy (i.e., more than one female in the group gave birth to pups), and duration (i.e., years) each breeding pair was bonded. I considered pair bond duration equal to 1 year at the first breeding event between two individuals. Sneaker male genotypes were reconstructed from mother and offspring genotypes using Program COLONY. Because I sampled wolves in early to mid-summer, pups were approximately 3 months old. I returned the following year to resample and determine whether they were alive or dead 15 months after birth (i.e., apparent survival). Fewer than 4% of wolves (approx. 1.1 pup/year total in the study areas) disperse within their first year in the study population^41^ thus I assumed absence at 15 months meant death. I further examined genotypes of harvested wolves throughout the state (250–350 wolves annually from mandatory check-in) to determine if pups dispersed early and were harvested. No early dispersal events were discovered this way. After extracting DNA from tissue of harvested wolves, I calculated annual harvest rate by dividing the number of genotypes detected in harvest in the study areas via tissue samples from mandatory harvest check-in by the number of genotypes detected the previous summer during field sampling. Finally, I used Program Genalex 6.503^32^ to calculate the observed heterozygosity of breeding individuals.

I used Program R^42^, package ‘lme4’, and generalized linear and mixed effects logistic regression models to test whether females chose mates with above average heterozygosity and to predict pup survival as a function of mated pair heterozygosity. To model female mate choice, I used a binary response variable with males of below average heterozygosity (0) and above average heterozygosity (1) against continuous female heterozygosity values. For pup survival (alive = 1, dead = 0), I also assessed factors previously found to influence survival of young, chiefly, number of adults in the group, number of years breeders were paired, harvest rate during the previous year (because harvest at time t affects pup survival at t + 1), and population density (wolves/1000 km^2^). I rescaled independent variables using a z-transformation for ease in comparing the resultant model coefficients. I considered a female’s mate choice as a unique event each year. However, because pairs often mate for > 1 year, I also modeled “mated pair ID” as a random effect using a mixed effects model. P-values for each covariate were estimated using asymptotic Wald tests from package ‘lme4’ in Program R version 4.0.4^42^. I used Akaike’s Information Criterion (AIC) to compare candidate and null models. Finally, I used a T-test to assess potential differences in heterozygosity between sneaker males and group-living mated males.

### Ethics approval

Sampling was conducted under University of Montana IACUC (Animal Use Protocol 008-09MMMCWRU).

## Results

I genotyped 925 individuals in 16 groups and estimated survival for 357 pups from 2008 to 2017. Individuals were detected 1–44 times. Average heterozygosity for all sampled individuals was 0.75 (SD = 0.11), 7% higher than the wolves reintroduced in 1995 and 1996 (H_o_ = 0.70^18^), albeit using several different loci. Across 83 independent reproduction events, I identified 37 female breeders, 30 male breeders, and 10 additional “sneaker” male breeders who appeared to be unaffiliated with sampled groups. Average breeding female and male observed heterozygosity was 0.74 (SD = 0.10) and 0.78 (SD = 0.12), respectively. Average heterozygosity for unsampled, “sneaker” male breeders was 0.65 (SD = 0.10), significantly lower than mated group-living males (*T* = 4.13, *P* = 0.0008). Across all mated pairs, average heterozygosity was 0.75 (SD = 0.07).

Females with below average heterozygosity did not choose mates with above average heterozygosity (β = − 0.43, SE = 2.63, *P* = 0.87). The model predicting female mate choice as a function of male heterozygosity performed poorly compared to a null, intercept only model (+ 1.97 AIC from null model). Median genetic relatedness between breeders was very low, 0.00 (range 0.00–0.54), although there were five instances (6%) of wolves breeding with related individuals ($\overline{x }$= 0.51, range 0.50–0.54) in their group. Mean relatedness of simulated mated pairs was 0.00 assuming random mating in the population.

Pup survival was not higher for mated pairs with above average heterozygosity in either fixed (β = − 0.12, SE = 0.12, *P* = 0.32; Table 1) or mixed effects models (β = − 0.01, SE = 0.19, *P* = 0.94; Table 1). The dominant variables predicting pup survival in fixed effects models were harvest rate (β = − 0.56, SE = 0.12, *P* < 0.0001) and years pairs were mated (β = 0.28, SE = 0.13, *P* = 0.04, Table 1). Only harvest rate was significant in mixed effects models (β = − 0.61, SE = 0.14, *P* < 0.0001, Table 1). The random effect for “pair ID” in the mixed effects model had a SD = 0.85 and likely captured, in part, the positive effect of years pairs were mated. Both fixed and mixed effects models performed better than a null, intercept only model (> − 28.0 AIC from null model), however, a mixed effects model was most supported overall (− 7.3 AIC from fixed effects model).

**Table 1:** Covariates from fixed and mixed effects models predicting wolf pup survival in Idaho, USA, 2008–2017.

Covariate	β	SE	*P*
**Fixed effects model**			
Intercept	0.02		
Harvest rate	− 0.56	0.12	< 0.0001
No. adults	0.19	0.12	0.11
Pair H_o_	− 0.12	0.12	0.32
Population density	− 0.18	0.12	0.13
Years paired	0.28	0.13	0.04
**Mixed effects model**			
Intercept	0.02		
Harvest rate	− 0.61	0.14	< 0.0001
No. adults	0.19	0.16	0.23
Pair H_o_	− 0.01	0.19	0.94
Population density	− 0.13	0.15	0.38
Years paired	0.26	0.19	0.17
RE (pair_ID)	0.85 (SD)		

*H*_*o*_ observed heterozygosity, *RE* random effect.

## Discussion

I show that genetic diversity remained high in a translocated population of wolves > 20 years after reintroduction. The mechanism for maintaining such diversity did not appear related to individuals preferentially choosing more genetically diverse mates. I found, however, that wolves generally (94%) did not mate with family members and appeared to select mates randomly with respect to genetic relatedness. Thus, inbreeding avoidance appears to be at least one mechanism maintaining genetic diversity in this population. Observed heterozygosity (0.75; range 0.50–1.00) was similar to that reported by Ausband and Waits (2020; 0.76^43^) for Idaho’s wolf population although their estimate did not include the 357 pups that I present here. The observed heterozygosity in Idaho’s reintroduced population of wolves was similar to values reported from other recovering wolf populations^44–46^. Roininen et al.^45^ reported observed heterozygosity of 0.68–0.71 for wolves in Finland through 2004, although more recent estimates through 2009 show heterozygosity has declined in the population (0.62) likely due to inbreeding and population isolation^44^.

How is high genetic diversity being maintained in Idaho’s wolf population? Some studies have shown that individuals will choose mates with higher average heterozygosity than what is expected if there were random mating in the population^13,14^. I did not observe such mate selection and it does not appear to be a mechanism for conserving genetic diversity in Idaho’s wolves. Idaho is part of larger population of wolves encompassing four other western states in the U.S. (Oregon, Washington, Montana, Wyoming) as well as the province of British Columbia in Canada. This larger western North America metapopulation holds > 3000 wolves^47–51^ and although movement of wolves from some areas into Idaho may be limited and unlikely, we do know wolves frequently disperse long distances crossing state and provincial boundaries in the population^41^. I posit that the high heterozygosity observed in Idaho reflects inbreeding avoidance and dynamics inherent to the larger western North America metapopulation and movement (and subsequent breeding) of wolves into and out of Idaho.

I hypothesized that females would choose mates who were more genetically diverse than average, particularly when the male was not affiliated with the group. Surprisingly, such sneaker males were not as genetically diverse as males who were mated within groups. Reid et al.^52^ found male song sparrows (*Melospiza melodia*) who were inbred rarely sired extra-pair offspring. While sneaker males in our study had lower heterozygosity than average (0.65), it was not appreciably lower than the heterozygosity of the original reintroduced wolves, albeit using several different loci^18^. Female wolves enter estrus once per year for a period of just one week^53^ and the availability of a potential mate must overlap this period. Females choosing to mate with a male who is unaffiliated with the group may be “making the best of a bad job” by avoiding inbreeding with a related male or infertile partner at the cost of breeding with a sneaker male who may not have optimal genetic diversity. Relatedness between breeding females and sneaker males was 0.07 (SD = 0.10), slightly higher than what I observed for group-living pairs (0.00). This suggests that sneaker males may be individuals who have dispersed from their natal group and subsequently exist nearby as “floaters” or as members of neighboring groups.

Ultimately, the genetic diversity metric I used was not predictive of wolf pup survival. Harvest and years pairs were mated were influential predictors of pup survival, mirroring previous work conducted on Idaho’s wolves^54,55^. Harvest has both direct (i.e., pups are hunted and trapped) and indirect effects on pup survival (i.e., harvest affects group composition thereby reducing pup survival). Additionally, pairs mated for longer periods of time appear to improve their ability to protect and provision young, ultimately leading to higher reproductive rate for such pairs^55,56^.

A lack of genetic diversity can result in lower reproduction and survival and ultimately increase the probability of extinction^6,8^. Although I did not observe Idaho’s wolves to be genetically impoverished, large reductions in population size or reduced connectivity with the larger western North America metapopulation could conceivably produce such effects. Rigorous genetic monitoring and tests for associations between diversity and reproduction might be useful in the event of large wolf population reductions.

## Data Availability

Data can be found at: 10.5281/zenodo.5764792.
